# Efficacy of *Salvia miltiorrhiza* injection combined with steroids in the treatment of oral submucous fibrosis: A meta-analysis of randomized controlled trials

**DOI:** 10.1097/MD.0000000000016339

**Published:** 2019-07-05

**Authors:** Hui Xie, Jincai Guo, Boyu Tan, Hao Wu

**Affiliations:** a Changsha Stomatological Hospital; b Hunan Provincial People's Hospital, Changsha, Hunan, China.

**Keywords:** meta-analysis, oral submucous fibrosis, *salvia miltiorrhiza*, steroids

## Abstract

**Background:**

This study was performed to systematically review the efficacy of *Salvia miltiorrhiza* injection combined with steroids in the treatment of oral submucous fibrosis.

**Methods:**

We searched 9 databases: Web of Science, PubMed, Medline, EBSCO, Embase, The Cochrane Library, WanFang Data, the China National Knowledge Infrastructure (CNKI), and Chinese Scientific Journals Full-text Database (VIP). Randomized controlled trials were collected to study the treatment of oral submucous fibrosis by *S miltiorrhiza* injection combined with steroids. Each database was searched from inception to November 2018. RevMan 5.3 software was used for the meta-analysis.

**Results:**

In total, 13 randomized controlled trials involving 1190 patients were included. The results of the meta-analysis showed that compared with conventional treatment, *S miltiorrhiza* injection combined with steroids could significantly increase the maximal mouth opening [mean difference (MD), 0.23; 95% confidence interval (CI), 0.16–0.30; *P* <.0001], decrease the oral mucosal lesion area (MD, −1.35; 95% CI, −2.46 to −0.25; *P* = .02), improve the subjective symptom burning sensation (MD, −0.77; 95% CI, −1.38 to −0.16; *P* = .01), and reduce adverse drug reactions (risk ratio, 0.27; 95% CI, 0.14–0.49; *P* <.0001).

**Conclusions:**

The results of this meta-analysis from current evidence showed that compared with conventional treatment, *S miltiorrhiza* injection combined with steroid could significantly improve the maximal mouth opening and the subjective symptom burning sensation as well as decrease the oral mucosal lesion area without increasing adverse effects.

## 1 Introduction

Oral submucous fibrosis (OSMF) is a chronic and insidious disease that is recognized as an oral precancerous lesion.^[1]^ OSMF mainly presents as difficulties in mouth opening and tongue movement, a burning sensation, and fibrosis of the oral mucosa that lead to dietary restrictions, impaired speech, and difficulty in maintaining oral health.^[2]^ Several factors may trigger the disease, including nut chewing, genetic predisposition, immunologic processes, ingestion of chili peppers, and nutritional deficiencies.^[3,4]^ Epidemiological evidence indicates that chewing betel nuts is one of the most important risk factors for OSMF.^[5–8]^ During the past few decades, the available treatments for OSMF such as physical therapy and medical and surgical interventions have varied; however, these treatments are largely ineffective.^[3]^ The drugs used to treat OSMF include steroids, enzymes, peripheral vasodilators, vitamins, antioxidants, and minerals.^[9,10]^ No drug can completely relieve the symptoms of OSMF; therefore, identification of an effective drug for OSMF is necessary.

In traditional Chinese medicine theory, OSMF pertains to “silt disease.” This term is primarily based on the special therapeutic method of promoting blood circulation to remove blood stasis. *S miltiorrhiza* injection (SMI) is a conventional drug in traditional Chinese medicine therapy and is very effective in activating blood circulation to dissipate blood stasis.

SMI has been recorded in the Chinese Pharmacopoeia since 1963 and is regulated as a common hemorheologic agent that promotes blood circulation, stops blood stasis, nourishes blood, and calms the nerves.^[11]^ SMI is made of the extraction of *Radix S miltiorrhiza*, called *Danshen* in Chinese. This extraction has been shown to improve symptoms of OSMF in many clinical randomized controlled trials (RCTs), but there is a lack of evidence-based medical data regarding its effectiveness. We conducted a meta-analysis to systematically evaluate existing clinical RCTs of SMI combined with steroids in the treatment of OSMF to generalize the application of SMI and provide a clinical reference for OSMF. The main purpose of this study was to evaluate the effect of SMI combined with steroids on alleviating the signs and symptoms of OSMF.

## 2 Methods

### 2.1 Data sources and search strategy

Nine databases [Web of Science, PubMed, Medline, EBSCO, Embase, The Cochrane Library, WanFang Data, the China National Knowledge Infrastructure (CNKI), and Chinese Scientific Journals Full-text Database (VIP)] were searched from inception to 30 November 2018. RCTs on the treatment of OSMF by SMI combined with steroids were collected. For example, the PubMed search strategy was as follows:

(1) “Oral submucous fibrosis” [MeSH]
(2) “Atrophia idiopathica mucosae oris” [Title/Abstract] OR “Diffuse oral submucous fibrosis” [Title/Abstract] OR “Submucous fibrosis of the palate and pillars” OR “Idiopathic scleroderma of the mouth” [Title/Abstract] OR “Asian sideropenic dysphagia” [Title/Abstract] OR “Sclerosing stomatitis” [Title/Abstract] OR “Idiopathic palatal fibrosis” [Title/Abstract] OR “Juxta-epithelial fibrosis” [Title/Abstract]
(3) ***(1)*** OR ***(2)***
(4) “Salvia miltiorrhiza” [Title/Abstract] OR “Danshen” [Title/Abstract]
(5) ***(3)*** AND ***(4)***

### 2.2 Study selection

Two reviewers (Jincai Guo and Boyu Tan) independently screened the literature, extracted the data, and checked each other's results. If their opinions differed, a third reviewer (HX) assisted in the judgment. If the data were incomplete, the author was contacted in an attempted to acquire the data. When screening the literature, the title and abstract were reviewed first. After excluding clearly unrelated literature, the full text was read, and whether to include the study was determined according to the inclusion and exclusion criteria.

### 2.3 Data extraction

The contents extracted from each of the selected studies mainly included basic information, including the first author's name and the publication year; baseline characteristics of the subjects, including sample size, age, and sex; specific interventions, including drug names, doses, frequency of use, and course of treatment; key elements of risk-of-bias assessment; and relevant indicators of treatment outcome.

### 2.4 Statistical analysis

Statistical analysis was performed using RevMan 5.3 software (Cochrane Collaboration, Copenhagen, Denmark). Continuous data were analyzed using the mean difference (MD) and 95% confidence interval (CI), while dichotomous data were analyzed using the risk ratio (RR) and 95% CI. Heterogeneity was evaluated by the chi-square test (α = 0.1) and the inconsistency index statistic (I^2^). If no heterogeneity was observed (*P* >.1, I^2^ ≤50%), a fixed-effects model was chosen. If statistical heterogeneity was present (*P* ≤.1, I^2^ >50%), further analysis of the source of heterogeneity was performed after excluding the effects of significant clinical heterogeneity, and a random-effects model was used for the meta-analysis. A funnel plot was employed to judge potential publication bias. Egger test and Begg test were also used to evaluate publication bias.

## 3 Results

### 3.1 Literature search

**Figure 1:**
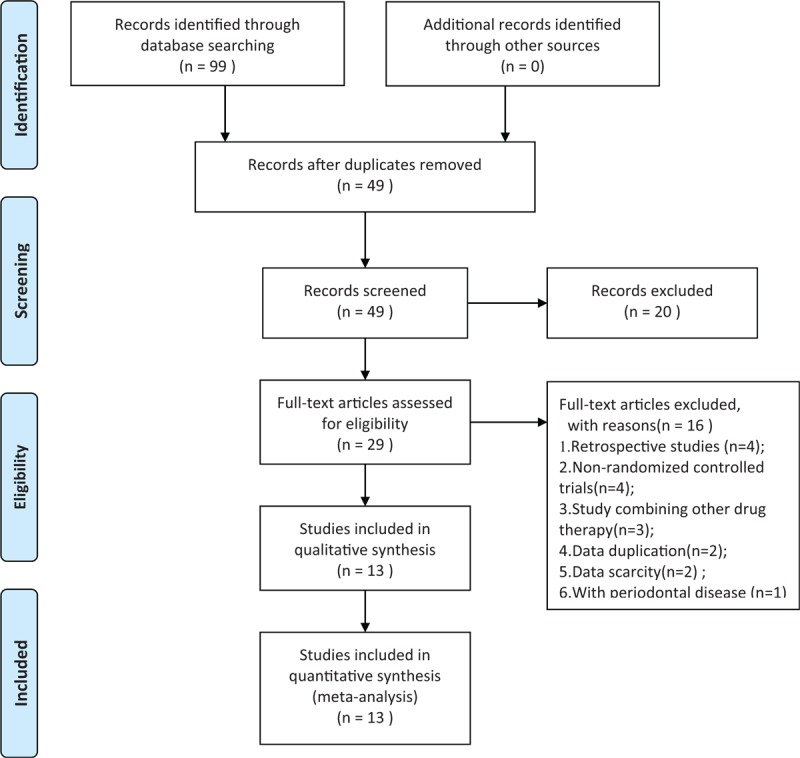
Flow chart of the study selection.

In total, 99 relevant studies were examined using our search strategy. Fifty duplicate studies were excluded. After screening of the titles and abstracts of the remaining 49 articles, 20 papers were found to be irrelevant (4 case reports and 16 reviews) and were excluded. After reading the full text of each article, 16 publications were removed (4 retrospective studies, 4 non-RCTs, 3 studies involving combination with other drug therapy, 2 studies with data duplication, 2 studies with data scarcity, and 1 study involving periodontal disease). Based on the inclusion and exclusion criteria, 13 RCTs published from 2008 to 2018 were ultimately included in the meta-analysis.^[12–24]^ All were Chinese studies. The detailed screening process is shown in Figure 1.

### 3.2 Study quality evaluation

**Figure 2:**
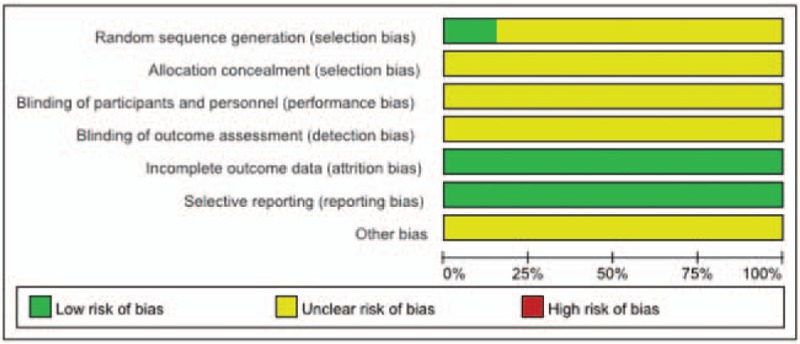
Risk-of-bias summary.

The risk of bias in the included studies was assessed by the Cochrane Handbook for Systematic Reviews of Interventions, Version 5.1.0. All included studies were clinical RCTs, but only 2 studies ^[17,22]^ introduced a specific method of randomized allocation (random number table); the others did not mention the randomization method used. No studies mentioned allocation concealment or double blinding. This suggests that the overall quality of the included studies was relatively low. The details of the study quality evaluation are shown in Table 1 and Figure 2.

**Table 1:**
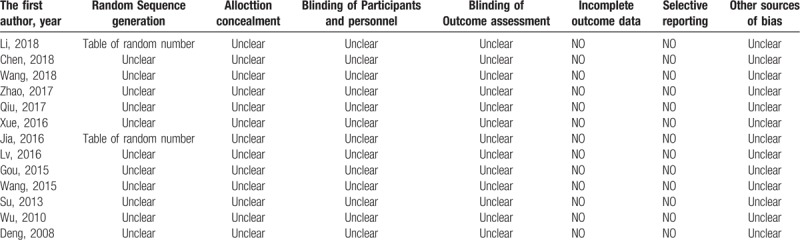
Quality assessment of included randomized controlled trials.

### 3.3 Data extraction

The characteristics of the selected studies are summarized in Table 2. The 13 studies were published from 2008 to 2018 and involved 1190 participants (606 in the trial group and 584 in the control group). In addition, OSMF affected men more often than women.

**Table 2:**
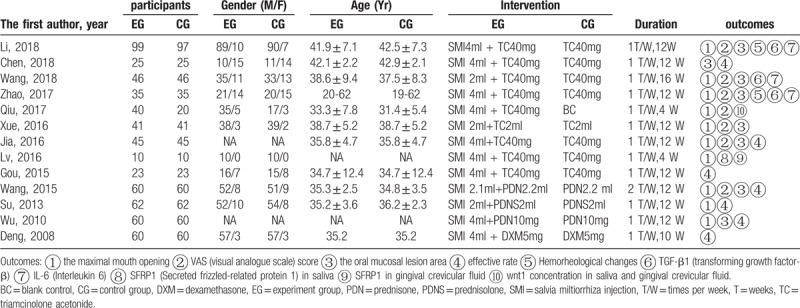
Characteristics of included studies.

### 3.4 Curative effect evaluation

In all 13 included studies, the main treatment effects of SMI combined with steroids on OSMF were the total effective rate, maximal mouth opening, oral mucosal lesion area, burning sensation, and adverse drug reactions.

#### 3.4.1 Total effective rate

**Figure 3:**
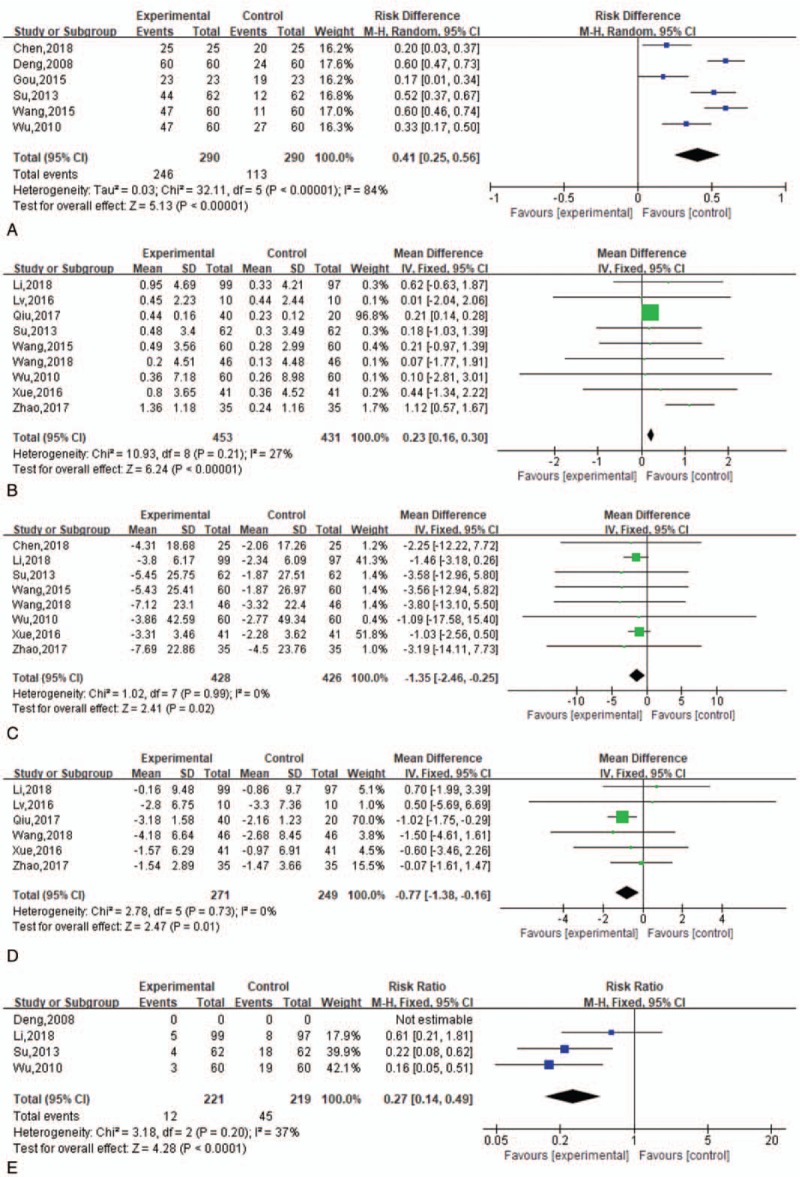
(A) Forest plot of total effective rate. (B) Forest plot of maximal mouth opening. (C) Forest plot of oral mucosal lesion area. (D) Forest plot of burning sensation. (E) Forest plot of adverse drug reactions.

Six studies involving 580 patients reported the total effective rate.^[12–16,24]^ Compared with conventional treatment, SMI combined with steroids had a significantly higher total effective rate (RR, 0.41; 95% CI, 0.25–0.56; *P* <.0001) (Fig. 3A).

#### 3.4.2 Maximal mouth opening

Nine studies involving 884 patients reported the maximal mouth opening.^[13,14,16,18–23]^ Compared with conventional treatment, SMI combined with steroids provided an increase in the maximal mouth opening (MD, 0.23; 95% CI, 0.16–0.30; *P* <.0001) (Fig. 3B).

#### 3.4.3 Oral mucosal lesion area

Nine studies involving 854 patients reported the oral mucosal lesion area.^[13,14,16,19–24]^ Compared with conventional treatment, SMI combined with steroids decreased the oral mucosal lesion area (MD, −1.35; 95% CI, −2.46 to −0.25, *P* =.02) (Fig. 3C).

#### 3.4.4 Burning sensation

Six studies involving 520 patients reported a burning sensation.^[18–23]^ A burning sensation was evaluated by the visual analog scale (VAS) score in these 6 studies. Compared with conventional treatment, SMI combined with steroids improved the subjective symptom of a burning sensation as shown by the VAS score (MD, − 0.77; 95% CI, −1.38 to −0.16; *P* = .01) (Fig. 3D).

#### 3.4.5 Adverse drug reactions

Three studies reported adverse drug reactions.^[13,14,22]^ Compared with conventional treatment, SMI combined with steroids reduced the incidence of adverse drug reactions (RR, 0.27; 95% CI, 0.14–0.49; *P* <.0001) (Fig. 3E).

### 3.5 Publication bias

**Figure 4:**
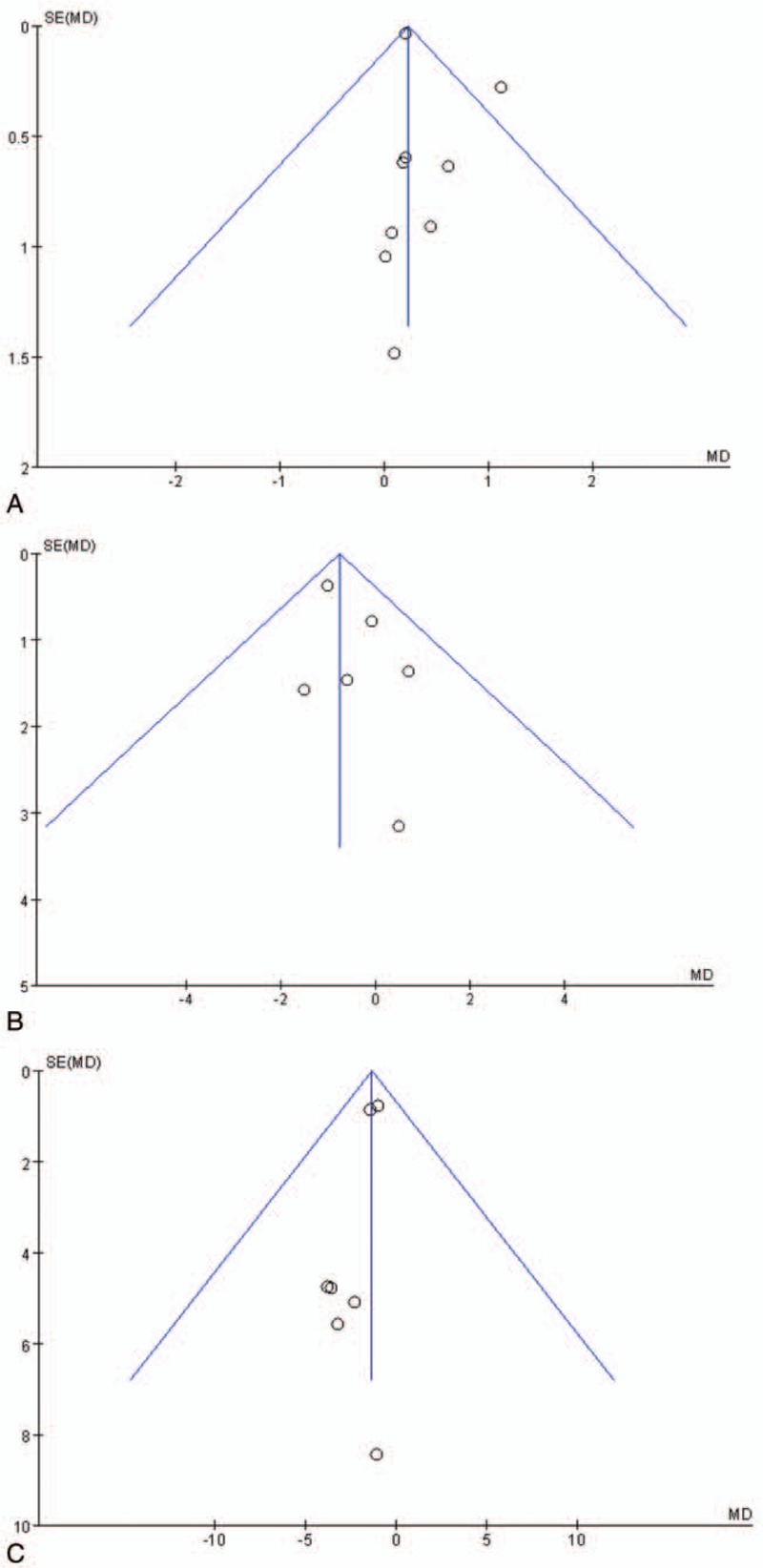
(A) Funnel plot of maximal mouth opening. (B) Funnel plot of burning sensation. (C) Funnel plot of oral mucosal lesion area.

According to the funnel plot of maximal mouth opening, a burning sensation, and the oral mucosal lesion area (Fig. 4A, B and C, respectively), asymmetric distributions were present on either side. These results suggest that the published literature might have a certain publication bias. However, the results of Egger test and Begg test regarding maximal mouth opening (t = 0.98, *P* = .98 and z = 0.73, *P* = .466, respectively), a burning sensation (t = −0.99, *P* = .38 and z = 0.00, *P* = 1.000, respectively), and the oral mucosal lesion area (t = 0.43, *P* = .679 and z = 0.37, *P* = .711, respectively) indicated no evidence of significant publication bias. The presence of publication bias could not be determined because fewer than 5 included studies reported adverse drug reactions.

## 4 Discussion

SMI is made of the extraction of *Radix S miltiorrhizae*. SMI is a traditional Chinese medicine with antioxidative, anti-inflammatory, antifibrotic, and antiapoptotic properties. It can improve fibrosis, increase superoxide dismutase activity, decrease malondialdehyde production, and upregulate the expression of matrix metalloproteinases 2 and 9.^[25,26]^ Salvianolic acid A, salvianolic acid B, and tanshinone IIA are important active compounds of SMI. All 3 active compounds have excellent antifibrotic activity in vitro. They can inhibit collagen accumulation and procollagen gene transcription. They can also inhibit activation of the mitogen-activated protein kinase/extracellular signal-regulated kinase pathway, Akt pathway, and transforming growth factor-β/Smad pathway.^[27]^

Steroids suppress or prevent inflammatory reactions. Steroids also act as immunosuppressive agents. They can prevent fibrosis by decreasing deposition of collagen and fibroblastic proliferation. Steroids such as dexamethasone, triamcinolone, betamethasone, and hydrocortisone have been used in the treatment of OSMF. They partly relieve the symptoms at an early stage of OSMF.^[28,29]^ Many studies on drug therapy for OSMF have involved steroids, such as hyaluronidase, chymotrypsin, and placental extract.^[30]^

Several studies have confirmed the efficacy of SMI combined with steroids in the treatment of OSMF, but evidence-based medical data are currently lacking. Thus, a meta-analysis is necessary to study the effects of SMI combined with steroids in the treatment of OSMF.

The results of this meta-analysis of current evidence show that compared with conventional treatment, SMI combined with steroids can significantly improve the maximal mouth opening and the subjective symptom of a burning sensation as well as decrease the oral mucosal lesion area without increasing adverse effects. SMI combined with steroids also seemed to improve the total effective rate based on our results, but the heterogeneity was high (I^2^ = 80%). The total effective rate does not have a unified standard, which is one of the most important causes of heterogeneity. Therefore, is not suitable to merge the data of the total effective rate.

Past studies have used different steroids such as triamcinolone acetonide, prednisone, prednisolone, and dexamethasone. Nine studies in the present meta-analysis used triamcinolone acetonide, 2 used prednisone, 1 used prednisolone, and 1 used dexamethasone. Through a subgroup analysis of triamcinolone acetonide and prednisone, we found that compared with conventional treatment, SMI combined with triamcinolone acetonide could significantly increase the maximal mouth opening (MD, 0.89; 95% CI, 0.43–1.34; *P* = .0001) and decrease the oral mucosal lesion area (MD, −1.29; 95% CI, −2.41 to −0.17; *P* = .02). SMI combined with prednisone could not significantly increase the maximal mouth opening (MD, 0.19; 95% CI, −0.90 to 1.29; *P* = .73) or decrease the oral mucosal lesion area (MD, −2.96; 95% CI, −11.11 to 5.19; *P* = .48). Thus, the subgroup analysis showed that SMI combined with triamcinolone acetonide more effectively improves symptoms of OSMF than does SMI combined with prednisone. SMI combined with triamcinolone acetonide is therefore recommended for the treatment of OSMF.

Although the exact mechanism of SMI in the treatment of OSMF is not entirely clear, many studies have suggested that it may be attributable to its antifibrotic, antioxidative, anti-inflammatory, and antiapoptotic effects. Therefore, it is reasonable to conclude that SMI may improve the maximal mouth opening and the subjective symptom of a burning sensation and decrease the oral mucosal lesion area in patients with OSMF through its antifibrotic, antioxidative, anti-inflammatory, and antiapoptotic activities.

SMI was generally well tolerated in terms of security, and adverse reactions were few in patients with OSMF. The main adverse reactions were acne, weight gain, fatigue, abdominal pain or back pain, and gastrointestinal discomfort. However, these conditions were relieved after symptomatic treatment with potassium, calcium, and gastric care solution, none of which affected the treatment of OSMF.^[13,14]^

The findings also showed a higher prevalence of chewing betel nuts among 15- to 49-year-old males, who are thus at higher risk of OSMF because chewing betel nuts is one of the most important risk factors for OSMF. Epidemiological evidence has shown that the prevalence of OSMF is 1.0% in Central China's Hunan Province (1.9% among men and 0.1% among women).^[7]^ However, no study has focused on the effects of age and sex on the efficacy of SMI combined with steroids in the treatment of OSMF. This is an important study direction in future.

In addition, the designs of the analyzed studies were not adequately stringent because they used a positive control rather than placebo. Moreover, the control group was administered different steroids.

Our analyses had some limitations. The medication was not identical in each experimental group and control group, and the included studies used a positive control rather than placebo. The overall quality of the included studies was low, and most of the studies did not specifically mention the methods of randomization, allocation concealment, and double-blinding; only 2 studies ^[17,22]^ specifically described these methods. This may have led to implementation bias and measurement bias. Additionally, all included studies were found in the published literature; no unpublished literature was included, which may have led to publication bias.

## 5 Conclusion

Our meta-analysis of 13 RCTs has shown that SMI combined with steroids is an effective treatment for improving symptoms in patients with OSMF. Symptom relief can significantly improve the maximal mouth opening and the subjective symptom of a burning sensation as well as decrease the oral mucosal lesion area without increasing adverse effects. However, existing studies still have some limitations. Future studies need to adopt correct randomization methods, implement allocation hiding, and apply blinding methods to improve the quality of the research methodology, reduce the risk of bias of research conclusions, and use more high-quality RCTs to further verify the research conclusions. The exact mechanism of SMI combined with steroids in the treatment of OSMF requires further study.

## Author contributions

Jincai Guo and Boyu Tan independently screened the literature and extracted and analyzed the data. If the opinions were different between Jincai Guo and Boyu Tan, Hui Xie assisted in the judgment. Jincai Guo proposed the preliminary concept of the study and drafted the article. Hui Xie, Boyu Tan, and Hao Wu read and approved the final manuscript.

**Formal analysis:** Jincai Guo, Boyu Tan.

**Methodology:** Boyu Tan.

**Software:** Jincai Guo.

**Writing – original draft:** Jincai Guo.

**Writing – review & editing:** Hui Xie, Hao Wu.
